# Predictors for long-term relapse of orthodontic treatment in patients with cleft lip and palate. A clinical follow-up study

**DOI:** 10.1007/s00784-024-05632-3

**Published:** 2024-04-03

**Authors:** Sarah Achterrath, Isabelle Graf, Romeo Guevara, Bert Braumann, Teresa Kruse

**Affiliations:** Department of Orthodontics, University of Cologne, Faculty of Medicine and University Hospital Cologne, Kerpener Str. 32, 50937 Cologne, Germany

**Keywords:** Unilateral cleft lip and palate, Bilateral cleft lip and palate, Long-term stability, Treatment outcome, Modified Huddart Bodenham Index

## Abstract

**Objectives:**

To identify predictors for long-term relapse of orthodontic therapy in patients with cleft lip and palate (CLP).

**Materials and methods:**

Patients with uni- and bilateral non-syndromal CLP were followed up at least two years after completion of their orthodontic therapy. Plaster casts of the start of treatment (T1), after completion of treatment (T2), and at follow-up (T3) were measured using the modified Huddart Bodenham Index. Characteristics of multidisciplinary therapy were taken from the patient files. Potentially influencing factors of relapse were investigated using logistic regression analyses and Spearman correlations.

**Results:**

In total 58.07% of the included 31 patients showed a stable treatment outcome at follow-up after an average of 6.9 years. Even if relapse occurred, 61.54% of these patients still showed improvement regarding their occlusion compared to baseline. Predictors for the occurrence of relapse were the severity of dysgnathia at baseline (*p* = 0.039) and the extent of therapeutic change (*p* = 0.041). The extent of therapeutic change was additionally a predictor for the extent of post-therapeutic relapse (*ρ* = 0.425; *p* = 0.019).

**Conclusions:**

Patients with CLP benefit from their orthodontic therapy in the long term despite an increased tendency to relapse.

**Clinical relevance:**

Results of this long-term study could be used to adapt the treatment concept for patients with CLP and reinforce the significance of a patient-centered orthodontic treatment concept for affected patients.

## Introduction

In Europe, non-syndromic cleft lip and palate (CLP) appears at a prevalence of approximately 1:1000 births [1]. Patients with CLP require multidisciplinary therapy, which is usually carried out at specialised centers. Among others, the disciplines involved include neonatology, orthodontics, oral and maxillofacial surgery, otorhinolaryngology, human genetics, speech therapy and psychology. Results of orthodontic therapy enable an increase in the patients’ quality of life [2, 3]. Nonetheless, due to the usually severe dysgnathia, orthodontic therapy of patients with CLP is complex and time-consuming. Therapy-associated difficulty with oral hygiene, potential discomfort and frequent check-ups can be stressful for patients [4, 5]. An increased tendency to relapse compared to patients without CLP might jeopardise the outcome of orthodontic therapy [6]. Studies of patients without CLP showed a relapse in 24.4% of patients 4–10 years after completion of orthodontic therapy [7]. Marcusson et al. followed up patients with unilateral CLP for an average of 5.6 years after completion of their orthodontic therapy and demonstrated relapse in 54% of patients [6]. In order to minimise a potential burden of care, a better understanding of orthodontic relapse in patients with CLP is needed.

Various factors influencing orthodontic relapse have been studied. Still, these studies mostly refer to patients without CLP: Kahl-Nieke et al. demonstrated the influence of initial malocclusion and extent of therapy on relapse [8]. Further, the beginning of therapy and the occlusion at the end of treatment were considered as crucial [9]. Another potential influencing factor is a post-therapeutic tension caused by the periodontal ligament and supra-alveolar fibres [10–12]. The retention protocol or persisting orofacial dyskinesia are also suspected of contributing to an orthodontic relapse [13, 14].

Factors influencing orthodontic relapse in patients with CLP are poorly understood. Especially the patients’ maxilla is at risk of relapse. The maxillary arch tends to show a post-therapeutic reduction of its transversal and sagittal dimensions [6, 15]. Palatal scar tissue probably compromises stability in patients with CLP [16]. In patients with unilateral CLP, Sumardi et al. identified the following factors for a long-term relapse: age at the end of treatment, gap closure in agenesis of the lateral incisor and occlusion at the end of treatment [17]. The existing literature concerning the pathogenesis of orthodontic long-term relapse in patients with uni- and bilateral CLP is incomplete. In order to minimise a potential burden of care, a better understanding of orthodontic relapse in patients with CLP is needed. Therefore, we performed a follow-up study of patients with uni- and bilateral non-syndromal CLP.

## Materials and methods

This study was approved by the ethics committee of the Cologne University’s Faculty of Medicine (No. 21-1131_1). Signed informed consent of patients was obtained before study participation. Inclusion criteria were: non-syndromal uni- or bilateral CLP, minimum age of 17 years at time of recruitment, completion of orthodontic therapy at least two years ago including the retention phase, fully documented multidisciplinary therapy, existing documentation models of start and end of treatment. Exclusion criteria were: documented syndrome involvement, minimum age not reached, orofacial clefts of smaller extent (cleft lip +/- alveolus), cleft palate only (CPO), facial clefts, currently undergoing orthodontic therapy, discontinuation of orthodontic treatment, completion of orthodontic treatment less than two years ago, duration of orthodontic treatment less than one year, incomplete documentation (plaster models or patient file). For patients who met the inclusion criteria alginate impressions were taken. Plaster models from the beginning of treatment (T1), completion of treatment (T2) and follow-up (T3) were measured using the modified Huddart Bodenham Index (MHB) [18].

### Evaluation of the patient file

The following information was extracted from patient files, pseudonymized and analyzed in Exel (Microsoft Exel 2019, Microsoft Corporation, Redmond, Washington, USA): date of birth, sex, LAHSHAL code, agenesis of permanent teeth, date of start of treatment, duration of treatment and appliances used, duration of treatment pauses, date and number of alveolar bone grafts, timing and number of osteotomies, retention appliances used.

### Measurements of the plaster models

**Fig. 1:**
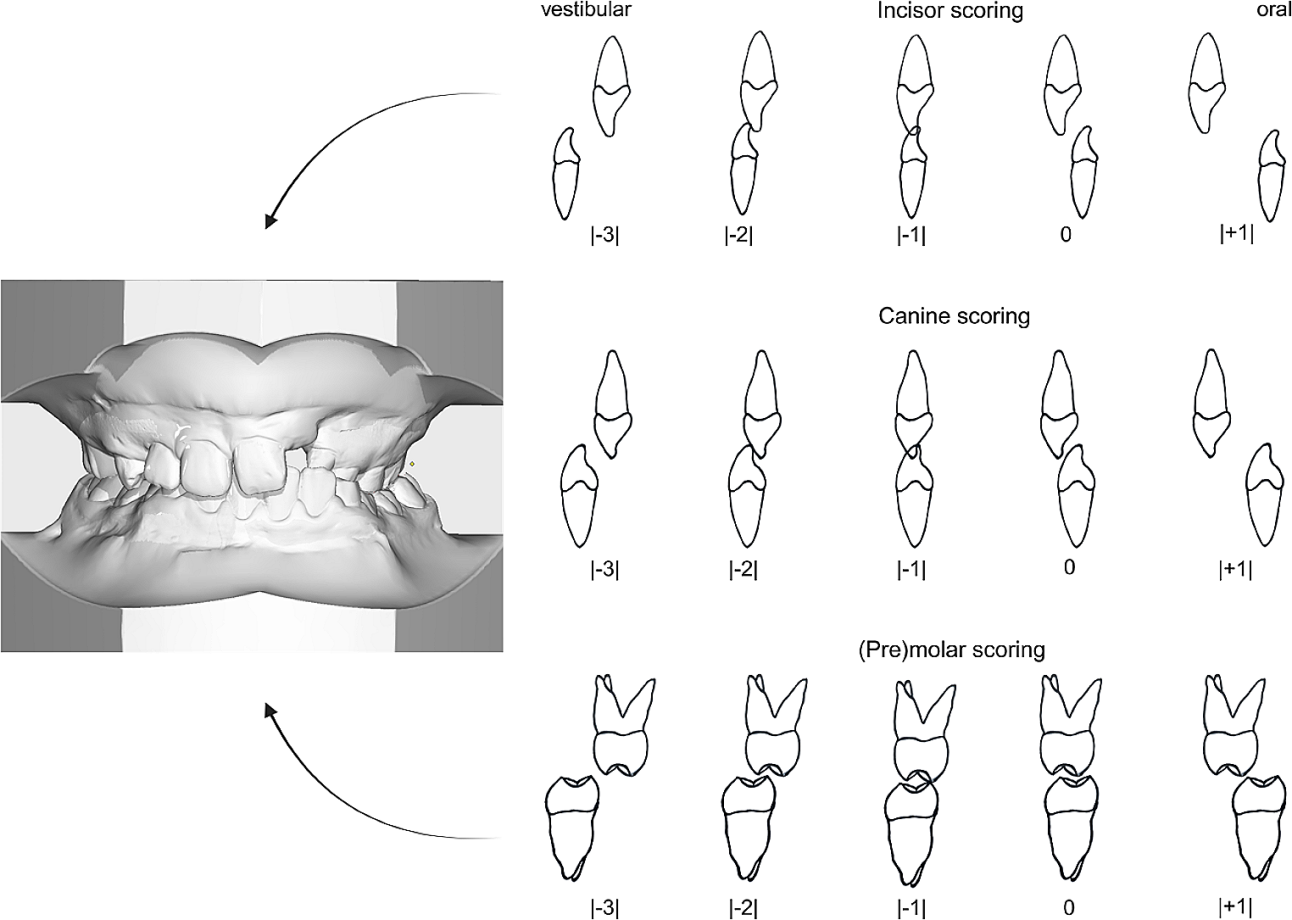
Adapted MHB measurements of the plaster models. The model shows the initial situation before treatment of a patient with unilateral CLP. Presentation of the measurement adapted from Noverraz et al. [20]

Plaster models were blinded and evaluated by one person. In general, the MHB Index is suitable for all cleft subtypes and all stages of dentition [19]. Antagonistic tooth pairs were evaluated according to their relation to each other (Fig. 1). The lateral incisors were not observed as they are often affected by agenesis [18].

Negative scores ( -1 to -3) were given if there was a relative deviation of the maxillary teeth towards the palate. Positive values (+ 1) were given for a vestibular deviation of the maxillary teeth. A physiological relation of the antagonistic tooth pairs was assigned a score of zero [20]. So far, the individual scores were added up to a total score [18, 20]. We modified this procedure and rated the individual values as amounts. Thus, no potential offsetting of positive and negative individual values might have possibly concealed existing dental malocclusions.

### Statistical analysis

Statistical analysis was performed using Microsoft Excel 2019, R 4.1 (R Core Team) and IBM SPSS Statistics 28 (IBM, Armonk, New York, United States). The following factors were defined as potentially influencing the probability of relapse: the cleft form, the number of tooth agenesis, extractions of permanent teeth during orthodontic treatment, gap closure in the absence of permanent upper incisors, the number of osteotomies, the |MHB| value at T1 and T2 as well as their difference (|MHB| ∆T1-T2) and, at T2, additionally a dichotomised variant (|MHB| = 0 vs. |MHB| > 0), the treatment duration, the summed duration of treatment pauses, the presence of an fixed retainer in the upper jaw, as well as the overjet and overbite at T1. Interactions of these factors were examined using Spearman correlations. Group comparisons for variables in the subgroups with and without relapse were conducted using the Mann-Whitney U test or the Chi-square test. Bivariate logistic regression analyses were used to examine each factor for predicting the occurrence of relapse. Further non-parametric Spearman correlations were used to analyse the correlations between the factors potentially influencing the relapse and the extent of the relapse (0 in patients without recurrence, otherwise increase in |MHB| values from T2 to T3).

## Results

### Sample

**Fig. 2:**
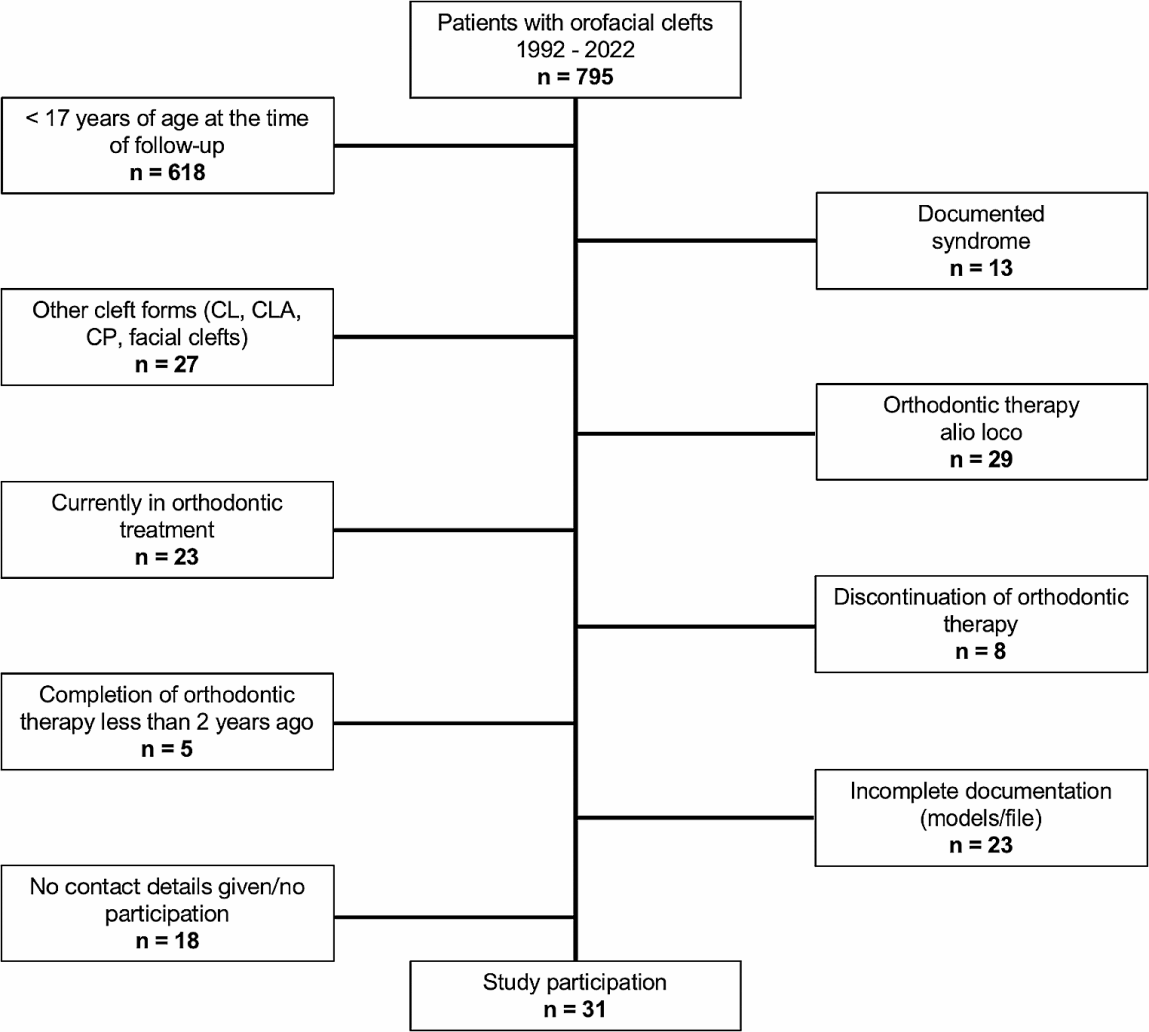
Flowchart of study participation

Out of 795 patients with orofacial clefts who consulted the Department of Orthodontics between 1992 and 2022, 49 patients met the inclusion criteria. A total of 31 out of 49 patients were willing to participate in this follow-up study (Fig. 2). Among these patients, 67.74% (*n* = 21) were men and 32.26% (*n* = 10) were women. Unilateral CLP was more frequent than bilateral CLP (74.20% vs. 25.80%). At the beginning of treatment, the patients were on average 7 +/- 2.8 years old. At the end of treatment, the patients were on average 17.9 +/- 3.0 years old. Follow-up took place at an average age of 24.9 +/- 3.9 years. In 45.16% of the patients, at least one permanent tooth was affected by agenesis. The lateral incisor on the left side was most frequently missing (25.81% of patients).

### Orthodontic treatment

All patients were treated by orthodontists who were familiar with the treatment of patients with CLP. In addition, all treatment steps were discussed in meetings of the treatment team at the Center for Rare Orofacial and Craniofacial Malformations of the University of Cologne. In total, 96.77% of all patients used removable appliances in the upper jaw. A transversal expansion in the maxilla using rapid maxillary expansion or quadhelix was performed in 74.19% of the patients. Maxillary protraction was not performed. All patients were treated with multibracket appliance. In 58,06% of patients, permanent teeth were extracted during treatment. Gap closure in the absence of permanent teeth in the upper jaw was performed in 29.03% of the cases. In 9.67% of patients, this involved the upper incisors. In addition, all patients received removable retention devices in both jaws. A fixed oral retainer was inserted in 22.58% in the maxilla and 58.06% in the mandible. The retention protocol included the use of a removable plate combined with either a fixed, stranded 6-point retainer or a fixed prosthetic restoration in the upper front. The fitting and consistent usage of these retention appliances were monitored over a period of at least one year. The orthodontic treatment of the patients lasted an average of 96.1 ± 29.5 months. Treatment with fixed appliances lasted an average of 55.8 months. For the group without extractions, the average duration was 55.8 months, and for the group with extractions 55.7 months. The mean value of treatment pauses in months was 32.52 ± 30.9.

### Surgical interventions during orthodontic therapy

All primary surgical interventions were performed by one experienced oral and maxillofacial surgeon. A total of 90.32% of patients underwent at least one alveolar bone graft. At the time of the first alveolar bone graft, the patients were on average 10.8 ± 2.9 years old. A second alveolar bone graft was necessary in 19.35% of patients. Le Fort I osteotomies, mono- and bimaxillary surgery and segment osteotomies were recorded under the term osteotomies. A total of 35.48% of patients required at least one osteotomy. More than one osteotomy was required by 9.68% of the patients. All osteotomies were performed after growth was completed. One patient had segmental distraction osteogenesis after completion of growth.

### Factors influencing relapse

In total, 58.07% of the patients had a stable long-term result of the treatment. Out of the patients with relapse, 61.54% showed persistent improvement compared to baseline. In the subgroup analysis, several characteristics proved to differ between both groups (Table 1). Accordingly, these traits were considered as factors potentially predicting relapse. These factors showed various statistically significant correlations among each other (*p* < 0.05). The comparison between groups showed statistically significant differences in the number of congenitally missing teeth (*r* = 0.85, *p* = 0.034) and the degree of overbite at T1 (*r* = 0.87, *p* = 0.029) (Table 1). In the bivariate logistic regression analysis, two factors had a statistically significant influence on the probability of relapse (*p* < 0.05) (Table 2). The amount of dysgnathia at the start of treatment (OR = 1.23; z-value = 2.06; *p* = 0.039) and the extent of therapeutic change (OR = 1.28; z-value = 2.04; *p* = 0.041) were predictors of relapse. The more severe the dysgnathia at the beginning of treatment and the more extensive the therapeutic changes were, the higher was the probability of relapse. The extent of therapeutic change also correlated statistically significantly with the extent of the relapse (*ρ* = 0.425; *p* = 0.019) (Table 3). The more extensive the changes were, the more pronounced the relapse was.

**Table 1:** Characteristics of the subgroups “stable” and “relapse”

	stable *n* = 18	relapse *n* = 13	group comparison
Unilateral cleft (percentage)	12 (66.7%)	11 (84.6%)	χ²(1) = 0.51, *p* = 0.477
Bilateral cleft (percentage)	6 (33.3%)	2 (15.4%)	χ²(1) = 0.51, *p* = 0.477
Number of congenital missing teeth (median value)	0.5	1	*r* = 0.85, *p* = 0.034*
Dysgnathia at baseline T1 [|MHB|] (median value)	|6.5|	|9|	*r* = 0.04, *p* = 0.364
Overjet T1 [mm] (median value)	2.25 mm	1 mm	*r* = 0.33, *p* = 0.177
Overbite T1 [mm] (median value)	2.5 mm	2 mm	*r* = 0.87, *p* = 0.029*
Extent of therapeutic change [|MHB|] (median value)	|5|	|8|	*r* = 0.02, *p* = 0.423
Gap closure for congenital missing upper incisors (percentage)	2 (11.1%)	1 (7.7%)	χ²(1) = 0.0, *p* = 0.999
Extractions of permanent teeth (percentage)	9 (50%)	9 (69.2%)	χ²(1) = 0.49, *p* = 0.483
Duration of treatment [months] (median value)	96.5 months	101 months	*r* = 0.30, *p* = 0.187
Treatment breaks [months] (median value)	19 months	17 months	*r* = 0.54, *p* = 0.109
Number of osteotomies (median value)	0	1	*r* = 0.06, *p* = 0.332
Occlusion at the end of treatment T2 [|MHB|] (median value)	|0|	|1|	*r* = 0.36, *p* = 0.165
Physiological occlusion at the end of treatment T2 [|MHB|] (percentage)	10 (56%)	3 (23%)	χ²(1) = 2.07, *p* = 0.150
Fixed retainer in the maxilla (percentage)	3 (16.7%)	4 (30.8%)	χ²(1) = 0.24, *p* = 0.623

^*^p-values represent statistical significance at 5% (p </= 0.05)

r: Pearson’s correlation coefficient

χ²: Chi-square test statistic

**Table 2:** Bivariate logistic regression analysis of factors potentially predicting the occurrence of relapse

	OR	z-value	*p*-value^*^
Cleft form	2.75	1.10	0.270
Number of congenital missing teeth	0.80	-0.83	0.407
Dysgnathia at baseline T1	1.23	2.06	0.039*
Overjet at baseline T1	0.90	-0.96	0.340
Overbite at baseline T1	0.96	-0.25	0.803
Extent of therapeutic change	1.28	2.04	0.041*
Gap closure for congenital missing upper incisors	0.67	-0.32	0.752
Extractions of permanent teeth	2.25	1.06	0.289
Duration of treatment	1.02	1.46	0.145
Treatment breaks	0.99	-0.51	0.610
Number of osteotomies	2.61	1.66	0.097
Occlusion at the end of treatment T2	1.04	0.41	0.679
Physiological occlusion at the end of treatment T2	4.17	1.76	0.079
Fixed retainer in the maxilla	2.22	0.92	0.360

^*^p-values represent statistical significance at 5% (p </= 0.05)

**Table 3:** Spearman correlations between the factors potentially influencing the relapse and the extent of the relapse

	ρ	*p*-value^*^
Cleft form	− 0.221	0.240
Number of congenital missing teeth	− 0.184	0.331
Dysgnathia at baseline T1	0.306	0.101
Overjet at baseline T1	− 0.207	0.272
Overbite at baseline T1	− 0.097	0.611
Extent of therapeutic change	0.425	0.019*
Gap closure for congenital missing upper incisors	− 0.209	0.267
Extractions of permanent teeth	0.338	0.068
Duration of treatment	0.220	0.243
Treatment breaks	− 0.034	0.857
Number of osteotomies	0.359	0.052
Occlusion at the end of treatment T2	0.109	0.566
Physiological occlusion at the end of treatment T2	− 0.268	0.152
Fixed retainer in the maxilla	0.038	0.172

^*^p-values represent statistical significance at 5% (p </= 0.05)

## Discussion

The aim of the present study was to identify factors that influence the likelihood of relapse of the orthodontic treatment outcome in patients with CLP. There are only few long-term studies in the literature on the stability of orthodontic treatment outcomes in patients with CLP [17]. Some studies mainly described the morphology of the relapse [6, 15]. Others investigated short-term relapse after the retention period [21, 22]. To our knowledge this study is the first to identify factors of long-term relapse in patients with unilateral and bilateral CLP.

The observed cohort can be considered representative of patients with cleft in the community. The ratio of male to female was 2.1 : 1 and the ratio of unilateral to bilateral CLP was 2.9 : 1. These distributions are consistent with the corresponding data in the literature [23, 24]. The prevalence of agenesis also corresponds to data from recent studies [24]. Results of the present study are thus transferable to patients of other centers with a comparable therapy concept.

The applied MHB Index for measurement has been used in previous studies to assess orthodontic treatment outcome in CLP [17, 25]. This index is sensitive, reliable, and objective. It has been shown to be superior to other indices [26, 27]. The metric scale is also well suited for statistical evaluation. As a disadvantage of the index only dental diagnoses are recorded. In addition, the vertical dimension is assessed neither skeletally nor dentally. The index focuses on transversal changes in the maxillary arch. Overall, the index is reproducible and also suitable for routine intraoral diagnostics in patients with CLP [28].

The model measurements revealed a stable treatment outcome in 58.07% of the patients after an average of 6.9 years. Marcusson et al. showed a relapse rate of 54% in patients with unilateral CLP after an average of 5.6 years [6]. Of these, 31% showed changes to the extent of one to two MHB points and 23% showed a relapse of more than four points [6]. Compared to the data in the literature and accounting for the increased tendency to relapse, the present cohort showed good occlusal stability. Factors influencing the occurrence of relapse were dysgnathia at baseline and the extent of therapeutic change. On the one hand, teeth and surrounding tissues tend to return to their pre-therapeutic position [29–31]. On the other hand, the reorganisation of the tissues and bone formation had already been completed at the time of the follow-up. Accordingly, this effect is unlikely to have influenced the relapse. Instead, the relationship between dysgnathia at the beginning of treatment and the occurrence of a longitudinal relapse could be due to the palatal scar tissue. In the present cohort, group comparisons revealed a statistically significant greater overbite at T1, which is more common in patients with bilateral CLP, in the relapse group. Pronounced scar tissue may have led to pronounced dysgnathia at baseline. The retractive potential of the scar tissue might persist even after the end of treatment and lead to a longitudinal relapse. In addition, studies of unoperated patients with CLP also showed transverse growth inhibition of the maxilla [32]. This underlines a potential intrinsic growth inhibition with a subsequent intrinsic tendency to long-term relapse. Nonetheless, Kahl-Nieke et al. described the association between dysgnathia at baseline and longitudinal orthodontic relapse already in patients without CLP [8]. Accordingly, explanations of this relationship must take other aspects into account. Since the dysgnathia at the beginning of treatment correlates with the extent of therapeutic change statistically significantly, it is questionable whether one of the two factors is an independent predictor. The influence of the extent of therapeutic change has been demonstrated in several studies in patients without CLP [8, 29, 33]. We were able to show that the extent of therapeutic change is an influencing factor of relapse in patients with CLP. Besides the scar tissue, the orofacial musculature could be responsible for this correlation [14]. Extensive therapeutic changes may complicate the adaption of the muscles, increasing the risk of relapse.

Patients with congenital agenesis of permanent teeth can be treated with either orthodontic gap closure or orthodontic space opening. This will affect the sagittal dimension, especially if the decision is to be made anteriorly. The decision to close the gap should always be based on the expected subsequent constriction of the dental arch, also for stability reasons. Sumardi et al. found that gap closure increased the risk of relapse in patients with unilateral CLP [17]. In our study, we found that the number of missing teeth was statistically significant higher in the relapse group. However, neither the number of missing teeth nor a past gap closure predicted the risk of relapse significantly in bivariate logistic regression analyses. Neither did those variables predict the strength of relapse. Consequently, based on the available data, the impact of these variables remains unclear.

Results of this follow-up study can be used to identify specific risk groups with an increased risk of long-term relapse. Patients with an increased risk of relapse require adapted patient information, therapy and follow-up.

Limitations of this study can be seen in the partly retrospective data collection. In addition, the number of cases limited the possibilities of statistical evaluation. Accordingly, it is not possible to conclusively determine factors for relapse on the basis of our data. Furthermore, the lack of assessment of the vertical dimension by the MHB Index might be seen as a methodological limitation.

Overall, prospective studies with larger case numbers are needed to explain orthodontic relapse in more detail.

## Conclusion

Patients with CLP benefit from their orthodontic treatment in the long term. Patients with comparatively severe dysgnathia at the beginning of treatment and extensive therapeutic changes are particular at risk of long-term relapse. Specialised centres should take this into account in their treatment concept.
